# BMS-754807 is cytotoxic to non-small cell lung cancer cells and enhances the effects of platinum chemotherapeutics in the human lung cancer cell line A549

**DOI:** 10.1186/s13104-016-1919-4

**Published:** 2016-03-01

**Authors:** S. Elizabeth Franks, Robert A. Jones, Ritesh Briah, Payton Murray, Roger A. Moorehead

**Affiliations:** Department of Biomedical Science, Ontario Veterinary College, University of Guelph, 50 Stone Road East, Guelph, ON N1G2W1 Canada

**Keywords:** BMS-754807, IGF-IR, Chemotherapy, Lung cancer, Proliferation, Apoptosis, Migration

## Abstract

**Background:**

Despite advances in targeted therapy for lung cancer, survival for patients remains poor and lung cancer remains the leading cause of cancer-related deaths worldwide. The type I insulin-like growth factor receptor (IGF-IR) has emerged as a potential target for lung cancer treatment, however, clinical trials to date have provided disappointing results. Further research is needed to identify if certain patients would benefit from IGF-IR targeted therapies and the ideal approach to incorporate IGF-IR targeted agents with current therapies.

**Methods:**

The dual IGF-IR/insulin receptor inhibitor, BMS-754807, was evaluated alone and in combination with platinum-based chemotherapeutics in two human non-small cell lung cancer (NSCLC) cell lines. Cell survival was determined using WST-1 assays and drug interaction was evaluated using Calcusyn software. Proliferation and apoptosis were determined using immunofluorescence for phospho-histone H3 and cleaved caspase 3, respectively.

**Results:**

Treatment with BMS-754807 alone reduced cell survival and wound closure while enhancing apoptosis in both human lung cancer cell lines. These effects appear to be mediated through IGF-IR/IR signaling and, at least in part, through the PI3K/AKT pathway as administration of BMS-754807 to A549 or NCI-H358 cells significantly suppressed IGF-IR/IR and AKT phosphorylation. In addition of BMS-754807 enhanced the cytotoxic effects of carboplatin or cisplatin in a synergistic manner when given simultaneously to A549 cells.

**Conclusions:**

BMS-754807 may be an effective therapeutic agent for the treatment of NSCLC, particularly in lung cancer cells expressing high levels of IGF-IR.

## Background

Lung cancer remains the leading cause of cancer-related deaths worldwide [1], and non-small cell lung cancer (NSCLC) represents ~80 % of the cases of lung cancer [2]. Lung cancer is often not diagnosed until later stages [3, 4] when it is no longer responsive to the standard treatment which is based on platinum chemotherapeutics and radiation [5–7]. Despite the development of a number of new targeted therapies against epidermal growth factor receptor (EGFR), vascular endothelial growth factor (VEGF), and anaplastic lymphoma kinase (ALK), overall survival rates remain poor [7–10]. There is a need to develop new treatment strategies including new drugs and understand the most effective way to incorporate them into current treatments. Several type I insulin-like growth factor receptor (IGF-IR) targeting agents have been in clinical development, including monoclonal antibodies and tyrosine kinase inhibitors, which are reviewed by Scagliotti and Novello [11] and Chen and Sharon [12]. Figitumumab, a monoclonal antibody against IGF-IR, reached a phase III clinical trial in non-adenocarcinoma NSCLC that was ended due to poor outcomes [13]. This disappointing outcome leaves researchers asking if a better understanding of the role of IGF-IR in lung cancer would identify certain biomarkers, histological subtypes, and drug combinations or sequences that could improve the effectiveness of targeting the IGF-IR as part of lung cancer treatment.

There is strong evidence implicating the IGF-IR in lung cancer. Molecular studies using lung cancer cell lines have shown that disruption of IGF-IR signaling will decrease proliferation, cell survival and migration in several lung cancer cell lines [14–16]. IGF-IR is also involved in tumor initiation since overexpression of the IGF-IR in type II alveolar cells or Clara cells was sufficient to induce spontaneous tumor formation in mice [17]. Several studies have found the IGF-IR to be frequently over-expressed in NSCLC, ranging from 30 to 84 % of patients [18–29]. While many of these studies did not find high IGF-IR expression to be associated with overall survival, it has been associated with progressive disease [18, 29–31], larger tumor size [23, 26], recurrence [24], and brain metastasis [32]. Additionally, high IGF-1 and low IGFBP-3 have been associated with poor outcome [33–39]. This suggests that increased activation of the IGF axis contributes to lung cancer progression; therefore, targeting the receptor would be an effective therapeutic strategy. Furthermore, activation of the IGF-axis via the IGF-IR has been identified as a mechanism of resistance to EGFR targeted therapies [40–45] and to cisplatin [46–48].

The IGF-IR is a tyrosine kinase receptor which exists as a dimer (α_2_β_2_): two monomers consisting of an alpha subunit and a beta subunit that are joined by disulfide bonds [49]. The IGF-IR signals through two main pathways, the PI3K/AKT pathway and the MAPK/ERK pathway [50, 51]. The IGF-IR will form heterodimers with the closely related insulin receptor (IR), which shares 60 % structural similarity [52–54]. The IGF-IR responds to ligand stimulation by IGF-I, IGF-II, and insulin. While the IR is primarily activated by insulin, it will also bind to IGF-II and can bind both IGF-I and IGF-II when part of a heterodimer with IGF-IR [55–60]. Since both IGF-I and IGF-II are reported to be important activators of IGF-IR signaling in lung cancer [61–64], there is a good rationale for targeting the IR as well as the IGF-IR in order to achieve complete inhibition of the IGF axis. Additionally, in breast cancer cells and Ewing sarcoma the IR was identified as a mechanism of resistance to IGF-IR specific monoclonal antibodies [65, 66]. There may be a therapeutic benefit to dual inhibition of both receptors over IGF-IR specific monoclonal antibodies. BMS-754807 is an ATP competitive inhibitor that targets both the IGF-IR and the IR [67, 68].

This study evaluated the efficacy of BMS-754807 alone, and in combination with cisplatin or carboplatin, on two NSCLC cell lines in vitro. Our findings showed that BMS-754807 significantly inhibited IGF-IR/IR, AKT and ERK1/2 phosphorylation and reduced the survival of both NSCLC cell lines evaluated. BMS-754807 significantly increase apoptosis in both A549 and NCI-H358 cells and significantly reduced proliferation in A549 cells. In A549 but not NCI-H358 cells, BMS-754807 induced synergistic cytotoxicity when combined with cisplatin or carboplatin. These findings demonstrate the benefit of inhibiting IGF-IR/IR signaling in a pre-clinical setting and suggest inhibiting IGF-IR/IR signaling should be further evaluated as a potential therapeutic strategy for the treatment of NSCLC.

## Methods

### Cell lines and reagents

Human lung cancer cell lines, A549 and NCI-H358, were purchased from American Type Culture Collection (ATCC, Manassas, VA). A549 and NCI-H358 cells were cultured in RPMI 1640 medium supplemented with 10 % FBS and 1 % antibiotic/antimycotic (v/v) (Life Technologies, Burlington, ON). Cell were maintained in incubators at 37 °C and 5 % carbon dioxide. Cell identity was confirmed through STR profiling (The Centre for Applied Genomics at The Hospital for Sick Children, Toronto, ON).

BMS-754807 was purchased from Sellek Chemicals (Houston, TX). Cisplatin and carboplatin were both purchased from Sigma-Aldrich (Oakville, ON). BMS-754807 was dissolved in DMSO (Sigma-Aldrich, Oakville, ON) and stored at −20 °C while cisplatin was dissolved in saline and carboplatin was dissolved in water. Cisplatin and carboplatin solutions were made fresh for each experiment, and stored at 4 °C for the duration of the experiment.

### Western blotting

Following treatment with BMS-745807, adherent cells were lysed, protein was isolated and western blots performed as previously described [69]. Primary antibodies were used against IGF-IRβ (1:1000; cat# 3027), phospho-IGF-IR (Tyr1135/1136)/IR (Tyr1150/1151) (1:500; cat# 3025), phospho-Akt(Ser473) (1:1000; cat# 4060), phospho-ERK1/2 (Thr202/Tyr204) (1:500; cat# 4377), and β-actin (1:5000; cat# 4970) (Cell Signaling Technology, Danvers, MA) overnight at 4 °C. An anti-rabbit secondary antibody (1:2000; cat# 7074) (Cell Signaling Technology, Danvers, MA) was used for 1 h at room temperature. Membranes were visualized using chemiluminescence substrate (PerkinElmer, Inc., Waltham, MA) and a FluorChem 8800 gel documentation system (Alpha Innotech—ProteinSimple, Toronto, ON) or a ChemiDoc XRS + imaging system (Bio-Rad Laboratories, Mississauga, ON). For western blots captured using the FluorChem 8800 gel documentation, densitometry of the bands was determined using AlpahEase FC software (Alpha Innotech—ProteinSimple, Toronto, ON) while western blots captured using the ChemiDoc XRS + gel documentation, densitometry of the bands was determined using ImageLab 5.2.1 software (Bio-Rad Laboratories, Mississauga, ON).

### WST-1 Assay

Cells were plated in a 96 well plate at a density of 2500 cells/well, and allowed to adhere for 4 h. BMS-754807, cisplatin, or carboplatin were added either alone or in combination or a solvent only control was added (0.1 % of total volume). Drugs and media were refreshed every 24 h. At 72 h, cell viability reagent WST-1 (Roche, Mississauga, ON) was added and absorbance was read using Universal Microplate Reader EL800 (Bio-Tek Insturments, Inc., Winooski, VT) after a 3 h incubation.

### Analysis of single and combination drug response

Cell viability determined with WST-1 (Roche, Mississauga, ON) assay after single agent and combination treatments with BMS-754807, cisplatin, and carboplatin were used to determine the IC_50_ and Combination Indices (CI) as calculated using Calcusyn software (Biosoft, Cambridge, UK). For combination effects: a synergistic effect is considered for a CI < 1; an additive effect is considered for a CI = 1; and an inhibitory effect is considered for a CI > 1.

### Immunofluorescence

Cells were plated on coverslips in 6-well dishes at a density of 0.5–1 × 10^5^ cells/well, allowed to adhere overnight, then treated with 0.5 μM of BMS-754807), or DMSO only (to 0.1 % v/v of total volume). Cells were fixed at 24 h post-treatment in 10 % buffered formalin (Fisher Scientific, Ottawa, ON) for 1 h. Cells were washed in PBS, permeabilized in 0.5 % Triton-X in PBS, and blocked in 5 % BSA in PBS. Then the cells were incuabated in primary antibodies against the proliferation marker Ki67 (1:400; cat# Ab15580) (Abcam, Toronto, ON) or apoptosis marker cleaved caspase 3 (1:200; cat# Ab3623) (Millipore, Etobicoke, ON) overnight at 4 °C. Cells were incubated with a fluorescent anti-rabbit secondary antibody (1:200; cat# A-21207) (Life Technologies, Burlington, ON) for 1 h at room temperature. Dapi nuclear stain (Life Technologies, Burlington, ON) was used as a counterstain, and coverslips were mounted using Prolong-gold (Life Technologies, Burlington, ON). Images were captured using Olympus BX61 microscope and Metamorph software (Molecular Devices, Sunnyvale, CA). Proliferation and apoptosis rates within cell populations were quantified by manual counts of the number of cells staining positive for Ki67 or cleaved caspase 3, respectively, and expressed as percentages relative to the total number of cells present as revealed by nuclear DAPI staining.

### Wound healing assay

Cells were plated on 12 well plates at a density of 2.0 × 10^5^ cells/well and cells typically reached 90–95 % confluency the following day. A wound was made through the centre of the well and cells were treated with 0.5 μM of BMS-754807, or solvent only (to 0.1 % v/v of total volume). Drugs and media were refreshed and wound closure was measured every 24 h. Images were captured using Olympus IX71 microscope and Q-Capture software (Q-Imaging, Surrey, BC).

### Statistics

All experiments were repeated in triplicate or quadruplicate. Statistical analysis was performed using Graphpad Prism 5 (Graphpad Software, Inc., La Jolla, CA). Means were compared using a one-way ANOVA and post hoc Dunnett’s test when multiple groups were compared back to a control value or a paired Student’s T test when two means were compared. Error is represented by Standard Error of Measurement (SEM). Statistical significance is noted as p < 0.05.

## Results

### IGF-IR Activity is Inhibited by BMS-754807

**Fig. 1:**
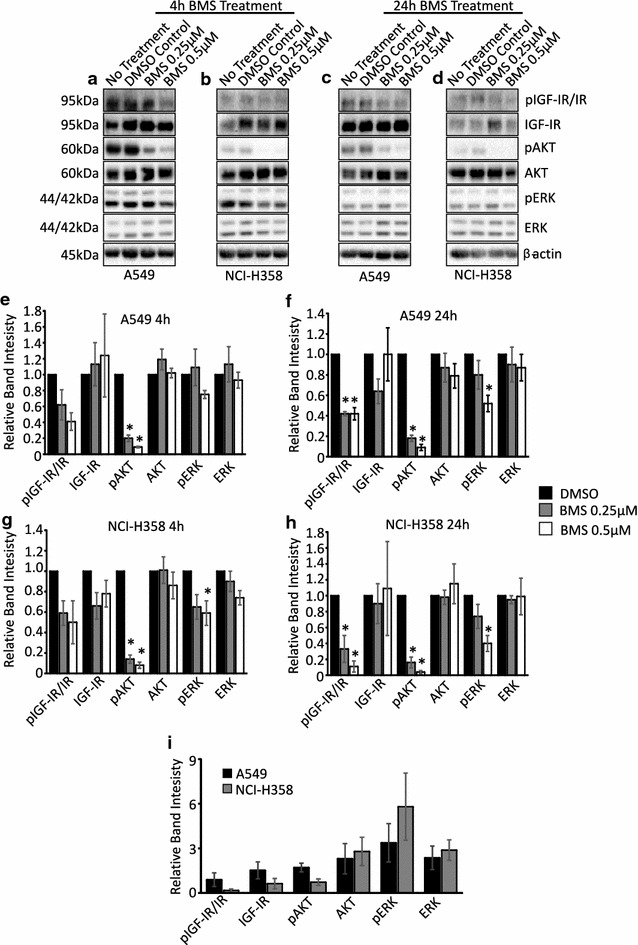
IGF-IR is expressed in lung cancer cells and its activity is inhibited by treatment with BMS-754807. Western blots of pIGF-IR/IR, IGF-IR, pAKT, AKT, pERK and ERK in A549 (**a**, **c**) and NCI-H358 (**b**, **d**) cells 4 (**a**, **b**) or 24 (**c**, **d**) hours after administration of 0.25 or 0.5 μM of BMS-754807. The *bar graphs* (**e**–**h**) represent the quantification of three independent western blots with the bars representing the means and the *error bars* representing SEM. The protein levels were normalized to the DMSO control group for each protein; the no treatment group was not quantified. β-actin was used as a loading control in the western blots and *p < 0.05 as determined by a Dunnett’s test. The bands from the 4 h No Treatment groups were quantified and are presented as the mean ± SEM for each protein relative to β-actin to illustrate the differences in protein levels in A549 and NCI-H358 cells (**i**)

A549 and NCI-H358 cells were treated with the dual IGF-IR/IR inhibitor BMS-754807 under regular growth conditions. When BMS-754807 was used at concentrations of 0.25 and 0.5 μM, the levels of activated IGF-IR/IR (pIGF-IR/IR) were reduced within 4 h, and a significant reduction in pIGF-IR/IR was observed at 24 h in both cell lines suggesting BMS-754807 inhibited IGF-IR/IR activation (Fig. 1a–h). Furthermore, treatment with BMS-754807 caused a concurrent decrease in phosphorylated AKT in both lung cancer cell lines (Fig. 1a–h). ERK phosphorylation was significantly reduced following administration of 0.5 μM but not 0.25 μM BMS-754807 (Fig. 1a–h).

To compare protein levels in A549 and NCI-H358 cells protein bands from the 4 h No Treatment groups were quantified. The IGF-IR protein was detected in both cell lines with A549 cells expressing more IGF-IR protein than NCI-H358 cells (Fig. 1a, b, i). A549 cells also possessed higher levels of phosphorylated IGF-IR/IR (pIGF-IR/IR) and phosphorylated AKT (pAKT) than NCI-H358 cells (Fig. 1a, b, i).

### Treatment with BMS-754807 inhibits proliferation and induces apoptosis

**Fig. 2:**
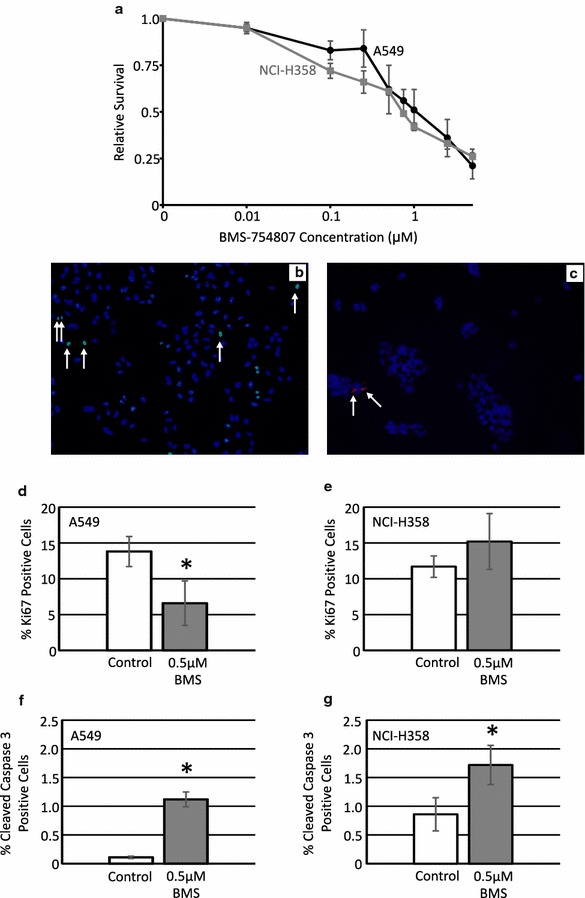
Treatment with BMS-754807 reduces cell viability in a concentration dependent manner. A549 (*black line*) and NCI-H358 (*grey line*) cells were treated with various concentrations of BMS-754807 every 24 h for 72 h. Cell viability was then measured with a WST-1 assay (**a**; n = 3). Immunofluorescence for Ki67 and cleaved caspase 3 were used to evaluate the impact of BMS-754807 on these cellular properties. A representative image of Ki67 (**b**; *green color*) and cleaved caspase 3 (**c**; *red color*) are presented as overlay images of cell nuclei stained with 4′,6-diamidino-2-phenylindole (DAPI, *blue color*). *Arrows* highlight some of the positive cells in each image. The number of Ki67 positive cells (**d**, **e**) and cleaved caspase 3 positive cells (**f**, **g**) along with the total number of cells were counted 24 h after treatment with 0.5 μM BMS-754807 and are presented as relative proliferation (**d**, **e**) or relative apoptosis (**f**, **g**) in A549 (**d**, **f**) and NCI-H358 (**e**, **g**) cells. The data is presented as mean ± SEM (n = 4) and the percentage of positive cells have been normalized to the DMSO control. *p < 0.05 as determined by a paired Student’s T-test

Concentration–response curves were generated to determine the sensitivity of each lung cancer cell line to BMS-754807. BMS-754807 administration was sufficient to reduce cell viability in both A549 and NCI-H358 cells (Fig. 2a). The IC_50_ values were determined using Calcusyn software (Table 1) to be 0.76 μM for NCI-H358 cells and 1.08 μM for A549 cells. In order to determine the specific cause of this reduction of cell viability, proliferation and apoptosis rates were determined in A549 and NCI-H358 cells following treatment with 0.5 μM BMS-754807 by immunoflourescence for Ki67 (Fig. 2b) or cleaved caspase 3 (Fig. 2c), respectively. BMS-754807 significantly inhibitied proliferation in A549 cells (Fig. 2d) but not NCI-H358 cells (Fig. 2e). Treatment with 0.5 μM BMS-754807 significantly increased apoptosis in both A549 (Fig. 2f) and NCI-H358 cells (Fig. 2g).

**Table 1:** IC_50_ concentrations for BMS-754805, cisplatin, and carboplatin

Cell line	Drug (µM)		
	BMS-754808	Cisplatin	Carboplatin
A549	1.08	3.33	65.29
NCI-H358	0.76	14.89	202.72

### BMS-754807 impairs wound closure

**Fig. 3:**
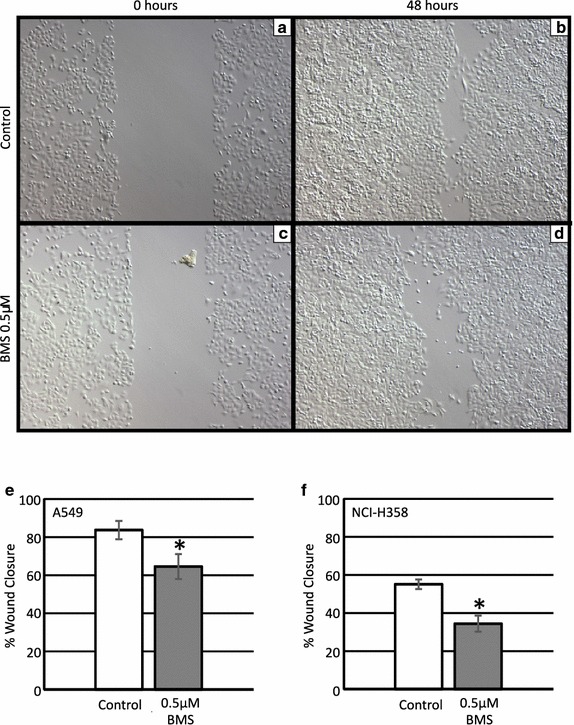
BMS-754807 reduces wound closure of lung cancer cells. Scratch wounds were made on near confluent plates of lung cancer cells. Cells were treated with BMS-754807 every 24 h, and wound closure was measured over 48–72 h. Representative images from A549 cells at 0 h (**a**, **c**) or 48 h (**b**, **d**) after treatment with either an appropriate concentration of DMSO or 0.5 μM of BMS-754807. Quantification of the relative scratch wound closure at 48 h in A549 (**e**) or at 72 h in NCI-H358 (**f**) cells was determined by measuring the remaining area not filled with cells and presenting this relative to the area of the scratch at time 0 h. Data represents mean ± SEM (n = 4), *p < 0.05 as determined by a paired Student’s T-test

To determine whether inhibition of IGF-IR/IR signaling influences wound closure, wound assays were performed following BMS-754807 treatment. Representative images of A549 cells shown at time 0 h (Fig. 3a, c) and 48 h (Fig. 3b, d) after treatment with the DMSO control (Fig. 3a, b) or 0.5 μM BMS-754807 (Fig. 3c, d). There was a minor, but statistically significant, impairment of wound closure in NCI-H358 and A549 cells treated with 0.5 μM BMS-754807 (Fig. 3e, f).

### Treatment with BMS-754807 or platinum chemotherapeutics reduces cell survival

**Fig. 4:**
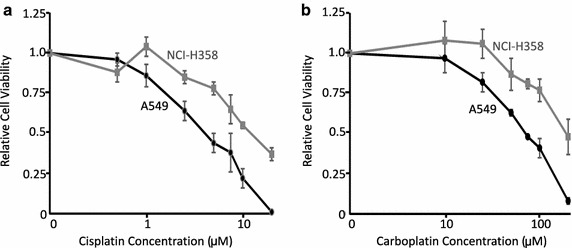
Treatment with platinum chemotherapeutics reduced cell viability in a concentration dependent manner. A549 (*black line*) and NCI-H358 (*grey line*) cells were treated with various concentrations of cisplatin (**a**), or carboplatin (**b**). Media and drugs were refreshed every 24 h for 72 h, then cell viability was measured with a WST-1 assay. All values are relative to the DMSO control and are presented as mean ± SEM (n = 3)

Concentration–response curves were generated to determine the sensitivity of each lung cancer cell line to cisplatin and carboplatin when they were used as single agents. Both cell lines were sensitve to growth inhibition by cisplatin (Fig. 4a) and carboplatin (Fig. 4b) in a concentration dependant manner. The IC_50_ values were determined using Calcusyn software (Table 1). A549 cells were more sensitive to platinum chemotherapeutics than NCI-H358 cells, with IC_50_ values of 3.3 and 14.9 μM for cisplatin and 65.3 and 202.7 μM for carboplatin, respectively. Both cell lines were more sensitive to cisplatin than to carboplatin as reflected by the lower IC_50_ values for cisplatin compared to carboplatin.

### BMS-754807 synergizes with platinum chemotherapeutics to reduce A549 Cell viability

**Fig. 5:**
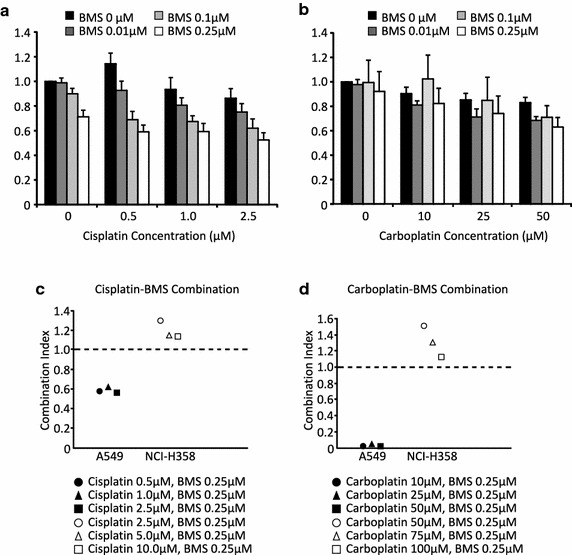
BMS-754807 synergizes with platinum based chemotherapeutics to enhance killing of A549 cells. Lung cancer cells were treated with a combination of BMS-754807 and either cisplatin (**a**, **c**) or carboplatin (**b**, **d**). Media and drugs were refreshed every 24 h for 72 h, then cell viability was measured using a WST-1 assay. Data illustrated in (**a**, **b**) are representative *graphs* of A549 cells treated BMS-754807 in combination with cisplatin (**a**) or carboplatin (**b**). This data is presented as mean ± SEM (n = 4). Combination indices were calculated using Calcusyn software and the data for cisplatin in combination with 0.25 μM of BMS-754807 is presented in (**e**) while the data for carboplatin in combination with 0.25 μM of BMS-754807 is presented in (**f**). A549 cells are plotted as black symbols while NCI-H358 cells are plotted as white symbols. The complete list of the interactions of all BMS-754807 concentrations with either cisplatin or carboplatin are presented in Tables 2 and 3

To determine if the addition of BMS-754807 could enhance the anticancer effects of platinum based chemotherapeutics at low concentrations, BMS-754807 was combined simultaneously with either cisplatin or carboplatin and the effects on cell viability were compared to those of each single agent. Three concentrations of BMS-754807 (0.01, 0.1, 0.25 μM in A549 cells and 2.5, 5.0, 10 µM in NCI H358 cells) were combined with three concentrations of cisplatin (0.5, 1, 2.5 μM) or carboplatin (10, 25, 50 μM in A549 cells and 50, 75 and 100 µM in NCI H358 cells), which based on the single agent concentrations were responsible for no more than approximately 25–30 % decrease in cell viability. At all concentrations of cisplatin and carboplatin used, the addition of BMS-754807 enhanced the effects of cisplatin and carboplatin (Fig. 5a, b). Combination indices were calculated using Calcusyn software to determine the nature of the interaction between cisplatin and BMS-754807 or carboplatin and BMS-754807. Representative plots of these interactions are presented in Fig. 5c, d. Combination indices <1 suggest synergistic interaction between the two drugs while combination indices of approximately 1 suggest additive drug interactions and combination indices >1 suggest antagonistic drug interactions. Based on the data presented in Fig. 5c, d, the combination of 0.25 μM BMS-754807 with cisplatin or carboplatin resulted in a synergistic reduction of cell survival in A549 cells (black symbols) but only additive or antagonistic interactions in NCI-H358 cells (white symbols). A complete list of the drug interactions for all the drug combinations tested are presented in Tables 2 and 3.

**Table 2:** Drug interaction between cisplatin and BMS-754807

Cell line	Cisplatin (μM)	BMS-754807 (µM)	Interaction^a^
A549	0.5	0.01	Synergistic
A549	1.0	0.01	Synergistic
A549	2.5	0.01	Synergistic
A549	0.5	0.1	Synergistic
A549	1.0	0.1	Synergistic
A549	2.5	0.1	Synergistic
A549	0.5	0.25	Synergistic
A549	1.0	0.25	Synergistic
A549	2.5	0.25	Synergistic
NCI-H358	2.5	0.001	Antagonistic
NCI-H358	5.0	0.001	Antagonistic
NCI-H358	7.5	0.001	Antagonistic
NCI-H358	2.5	0.1	Additive
NCI-H358	5.0	0.1	Additive
NCI-H358	7.5	0.1	Additive
NCI-H358	2.5	0.25	Antagonistic
NCI-H358	5.0	0.25	Additive
NCI-H358	7.5	0.25	Additive

^a^Combination index <1 = synergistic interaction, combinations index ~1

= additive interaction, combination index >1 = antagonistic interaction

**Table 3:** Drug interaction between carboplatin and BMS-754807

Cell line	Carboplatin (µM)	BMS-754807 (µM)	Interaction^a^
A549	10	0.01	Synergistic
A549	25	0.01	Antagonistic
A549	50	0.01	Synergistic
A549	10	0.1	Synergistic
A549	25	0.1	Additive
A549	50	0.1	Synergistic
A549	10	0.25	Synergistic
A549	25	0.25	Synergistic
A549	50	0.25	Synergistic
NCI-H358	50	0.001	Antagonistic
NCI-H358	75	0.001	Antagonistic
NCI-H358	100	0.001	Additive
NCI-H358	50	0.1	Antagonistic
NCI-H358	75	0.1	Antagonistic
NCI-H358	100	0.1	Additive
NCI-H358	50	0.25	Antagonistic
NCI-H358	75	0.25	Antagonistic
NCI-H358	100	0.25	Additive

^a^combination index <1 = synergistic interaction, combinations index ~1

= additive interaction, combination index >1 = antagonistic interaction

## Discussion

The IGF-IR is frequently expressed at high levels in NSCLC and thus represents a potential therapeutic target. One compound that targets IGF-IR (as well as IR) is BMS-754807 and this drug was evaluated in two human non-small cell lung cancer cell lines, A549 and NCI-H358. Both A549 and NCI-H358 cells express IGF-IR protein and the IGF-IR is phosphorylated in both cell lines under normal growth conditions with A549 cells containing higher levels of IGF-IR and phosphorylated IGF-IR than the NCI-H358 cells. BMS-754807 signficantly reduced, IGF-IR/IR, AKT and ERK phosphorylation in both cell lines with the most dramatic effect being the reduction in AKT phosphorylation. When Wittman et al. [67] first reported the development of BMS-754807 they reported it to be more effective at ERK inhibition than AKT with IC_50_ of 13 and 22 nM, respectively. However, subsequent reports in cell lines suggest the BMS-754807 is a more potent inhibitor of AKT than ERK phosporylation [68] which is consistent with our results. Furthermore, BMS-754807 was also reported to preferentialy inhibit the AKT pathway in breast cancer [70] and prostate cancer cells [71].

As a single agent, BMS-754807 effectively suppressed the survival of both A549 and NCI-H358 cells. This decrease in survival likely results from a significant increase in apoptosis induced by BMS-754807 in both A549 and NCI-H358 cells. BMS-754807 also significantly reduced proliferation in A549 but not NCI-H358 cells. This is the first report of the effects of BMS-754807 on survival, proliferation and apoptosis in A549 and NCI-H358 cells. BMS-754807 action has been evaluated in several other human NSCLC lines with similar IC_50_ values (0.5–5 µM range) however proliferation and apoptosis were not specifically assessed [68]. Another novel finding of this study was that BMS-754807, as a single agent, could suppress wound closure. Since several studies have demonstrated an important role for IGF-IR in the motility and invasion of lung cancer cells [72–76] it is possible that BMS-874807 can negatively impact lung cancer cell migration and potentially metastais. However, this finding of reduced wound closure requires confirmation as alterations in proliferation and apoptosis can influence the rate of wound closure independent of migration.

Platinum-based chemotherapeutic agents are commonly employed in the treatment of unresectable NSCLC [77]. Therefore, we evaluated the efficacy of cisplatin and carboplatin in A549 and NCI-H358 cells and found that both cell lines were more sensitive to cisplatin than carboplatin. This is consistent with the study by Mahalingham et al. [8] that reported cisplatin was more effective than carboplatin in the treatment of NSCLC. To evaluate whether BMS-754807 could enhance the effects of platinum chemotherpeutics, A549 and NCI-H358 cell survival was assessed following simultaneous administration of either BMS-754807 and cisplatin or BMS-754807 and carboplatin. In almost all the combinations tested, administration of BMS-754807 with cisplatin or carboplatin resulted in a syngersitic reduction in cell survival in A549 cells. In NCI-H358 cells, combinations of BMS-754807 with cisplatin or carboplatin resulted in additive or antagonistic drug interactions with respect to cell survival. It remains unclear why the combinations of BMS-754807 with the platinum agents was more effective in the A549 cells. A549 cells express higher levels of IGF-IR than NCI-H358 cells and thus it is possible that A549 cells are more reliant on IGF-IR signaling for proliferation and survival. It is also possible that the intrinsic insensitivity of NCI-H358 cells to platinum chemotherapeutics (IC_50_ for both cisplatin and carboplatin were considerably higher for NCI-H358 cells compared to A549 cells) impacted the interactions at the concentrations evaluated. A final potential explanation is that the different mutational profile of the two cells could influence drug interaction. While both cell lines harbour a mutation in *K*-*ras*, A549 cells have a mutation in *Cdkn2a* but contain wild type *p53* while NCI-H358 express mutant *p53* but wild type *Cdkn2a* (atcc.org). The only other study evaluating BMS-754807 in combination with chemotherapy in NSCLC found that BMS-754807 in combination with gefitinib resulted in synergistic reduction in cell survival in the human NSCLC cell line, NCI-H292 [78]. In small cell lung cancer (SCLC) targeting the IGF-IR using the monoclonal antibody NVP-ADW742 sensitizes SCLC cell lines to the effects of etoposide and carboplatin [79].

## Conclusions

In summary, this research demonstrates for the first time, the efficacy of BMS-754807 as a single agent in A549 and NCI-H358 cells and in combination with platinum-based chemotherapeutic agents in A549 cells. Therefore, BMS-754807 may be an effective therapeutic agent for the treatment of lung cancer, particularly in patients with lung tumors expressing high levels of IGF-IR.
